# Ovarian tissue autotransplantation improves longevity in mice

**DOI:** 10.3389/fphys.2024.1443494

**Published:** 2024-08-29

**Authors:** Nikolai N. Ruhliada

**Affiliations:** ^1^ Russian State Pediatric University, St.Petersburg, Russia; ^2^ St.Petersburg Emergency Medicine Institute by Djanelidze I.I, St.Petersburg, Russia

**Keywords:** longevity, lifespan, transplantation, ovaries, estradiol

## Abstract

In this study, we show the improvement in life longevity in an experimental mouse model after step-by-step autologous ovarian transplantation and compare its effects with exogenic transdermal estradiol usage. This has proven to be more efficient than “traditional” hormonal replacement therapy. Despite the high speed and effectiveness of estradiol replacement deficiency in blood by its oral or transdermal use, no significant increase in the life longevity of animals and possibly in women was noted. The function of the transplanted fragment is usually limited to 6–12 months. This is enough for oncofertility purposes, sometimes, but not for longevity improvement. We performed periodical tissue return (autologous transplantation), containing both the cortex and medulla in the experimental mouse model, which resulted in a statistically reliable improvement in longevity. Our experience indicates the important role of medullary ovarian factors in slowing the aging process in the body and increasing the life expectancy in the experiment. As shown, the transdermal estrogen supportive therapy for ovarian deficiency improves estrogen levels but causes much slower decreases in the follicle stimulating hormone (FSH) and luteinizing hormone (LH). Moreover, we attained the best longevity with step-by-step periodic ovarian autotransplantation, thus making “prosthetics” of ovarian function longer than it is preplanned physiologically [direct correlation between the levels of FSH and lifespan (r = 0.98)]. The experimental model we suggested could be projected to other mammals or humans as cortical transplantation provides the same results for reproduction restoration in mice and humans and even for hormone level normalization, but there is still a lack of information about anti-aging factors in the ovarian medulla and cortex. Hence, we consider that the most important factor for the anti-aging ovarian transplantation technology is to preserve and transfer both the medulla and cortex as parts of the whole ovary.

## Introduction

Ovarian function is one of the important factors in protecting the female body from aging (Arantes-Oliveira et al., 2002; Benedusi et al., 2015), as shown in the experimental female mouse model (Arantes-Oliveira et al., 2002; Kenyon et al., 1993; Cargill et al., 2003). Castration leads to a significant decrease in the life expectancy of individuals in comparison with uncastrated animals (Benedusi et al., 2015). The reason for this is both the protective angiogenic function of estrogens, which are manifested by cardio- and vascular-protective effects, and a number of biological substances found in the medullary layer of ovarian tissue, which prolong the life expectancy in mice, as observed in experiments with whole ovarian transplantation with depleted germ cells and the remaining germinal function (Habermehl et al., 2019; Deansley, 1954). In other experiments, authors achieved the abovementioned by pre-administering 4-vinylcyclohexene diepoxide (VCD) to mice, which caused complete atrophy of the pool of primordial follicles (Rivera et al., 2009).

One of the mechanisms of aging is the progression of cardiovascular diseases and excessive oxidation, resulting from postmenopausal changes in the endocrine balance. Pathophysiologically persistent metabolic disorder, which is also observed in the aging process, leads to the development of systemic inflammation and disruption of functions of all systems and organs in the body (Soleimani et al., 2011a). Circulating lipids and inflammatory mediators interact with each other at several levels, thereby exacerbating the development of chronic diseases, promoting morbidity. Cholesterol and modified lipids can directly activate inflammatory pathways (Soleimani et al., 2011a; Mason and Libby, 2015).

The attempts for autotransplantation of ovarian tissues have proved the possibility of maintaining both spontaneous ovulatory and endocrine functions in women, widely used in oncofertility cases. However, the time-limited functioning of transplanted grafts (usually no more than a year) meets the goal to save follicle growth, but it is not enough to compensate the hormonal deficiency and slow down the aging processes (Soleimani et al., 2011a; Bartke and Turyn, 2001; Wallace and Kelsey, 2010). At the same time, the exogenous administration of estrogens eliminates only part of the effects that develop during estrogen deficit/castration/in menopause. That is why menopausal hormone therapy (MHT), which is based on the exogenic administration of estrogens, does not significantly increase the life expectancy in women, despite the improvement in the quality of life (Cargill et al., 2003; Canderelli et al., 2007; Yoshida et al., 2011). Thus, in our experimental model, we considered and performed delayed step-by-step autotransplantation every 3 months to support the triggering and endocrine effects of autotransplanted fragments.

## Materials and methods

In order to clarify and compare the role and effects of ovarian factors after ovarian tissue autologous transplantation with MHT, we created a study design to assess the effect of MHT, castration, and various options for autotransplantation of ovarian tissues on the lifespan of mice and the dynamics of transplant hormonal activity (Demeestere et al., 2003; Donnez et al., 2013; Hikabe et al., 2016). For the experiment, we selected inbred female mice (c57BL/10) weighing 20–25 g at the age of 8 months. The protocols for animal experiments were approved by the Animal Experimental Ethics Committee of the Russian State Pediatric University (Approval No. 1456/2019) on 12 February 2019, in compliance with the National Institutes of Health guidelines for the care and use of laboratory animals. The present study followed international, national, and/or institutional guidelines for humane animal treatment and complied with relevant legislations from the Arrive International guidelines.

None of the animals were exposed to/were in the presence of males—they were separated at the age of less than 20 days. Mice of the c57BL/10 strain usually become reproductively competent between 45 and 60 days of age and reproductively senescent between 10 and 12 months of age. Ovariectomy at an average of 250–255 days of age will assure the influence that the female gonads might have in addition to the direct effects of gonadal hormones; thus, we prepared the model of early aging (Hikabe et al., 2016; Gougeon, 1996). Reproductive decline in c57BL/10 mice usually begins with irregular cycles at 8–10 months of age. Surgical procedures are most often conducted in an open field (exteriorizing the tract) and under a dissecting microscope (ovariectomy and transplant procedures). To obtain the ovarian tissue fragments in diestrus and the beginning of proestrus stages, we evaluated vaginal smears for the exact cycle stage (typical stringy mucus in which are entangled many leucocytes and a few nucleated epithelial cells, no large cornified cells (squamous) with degenerate nuclei). Metestrus and diestrus are homologous to the early and late secretory stages of the human reproductive cycle, respectively, characterized by high levels of progesterone.

Animal experiments were carried out in accordance with the general principles of experiments and the rules of laboratory practice in the Russian Federation (2003), as well as the provisions of the “European Convention for the Protection of Vertebrate Animals used for Experimental and other Scientific Purposes,” Strasbourg, France, 1985. All surgeries were performed under aseptic conditions, subject to mandatory general anesthesia during spontaneous breathing. This protocol describes the procedure used during the technique of ovary removal and autologous transplantation of ovarian fragments. After weighting and intraperitoneal anesthesia (I.P., 27-ga needle) injection, by using a cocktail of ketamine, xylazine, and acepromazine (65 mg/kg ketamine, 13 mg/kg xylazine, and 2.0 mg/kg acepromazine; cocktail dose—6.5 mL/kg), we returned the animal to the heated home cage until the anesthesia has taken effect. Once anesthetized, the animal was removed from the home cage and placed on heated paper towels. Using clippers (#40 blade preferred), the hair was removed a few mm lateral to the midline on each side, starting just below the ribs and moving distally, preparing a clipped “patch” approximately 2–3 cm^2^ on one side of the prone mouse, leaving a strip of hair approximately 1–2 cm covering the midline. The clipped areas were wiped with 70% ethanol, and betadine solution was applied to the clipped site using a cotton-tipped swab, while the antibiotic gel was applied for eye protection. The animal was placed in the prone position on a heated sterile surgical field, and the clipped area was wiped with 70% ethanol. A 1.0–1.5-cm paralumbar incision was made, and the skin was bluntly dissected from the underlying fascia. In the autotransplantation step, we do not open the fascia; the fragment of the defrozen ovary was fixed directly to the fascia using 4–5 sutures Prolene 7/0, and the skin was then closed using 2 Vicril intradermal sutures 5.0. In the case of bilateral ovariectomy, we opened the fascia, located the adipose tissue that surrounds the ovary in the abdominal cavity, gently extracted the fat pad and ovary, identified the ovarian bursa, grasped it using two microsurgical forceps, and made an incision in the ovarian bursa opposite the ovarian hilum to expose the ovary. After ovary removal, immediately start the xenon cryopreservation protocol for the ovary, each divided by six equal fragments for further autologous return. To obtain the tissue suspension under a dissecting microscope, we minced ovarian fragments to pieces, smaller than 0.2 mm^3^, by using a microblade and microsurgical forceps in saline media (usually 8–10 microfragments for each dose). The mechanically disaggregated tissue suspension was cryopreserved with the same xenon slow freezing protocol (Shishova and Fesenko, 2015; Isachenko et al., 2016) in a volume of 0.5 mL saline; thus, we obtained the dose for single injection for mice in group 3, further injected subcutaneously via a needle gauge 14. After the surgical step, we placed the animal in a heated recovery cage shaded from light and monitored the animal continuously until recovery from anesthesia. Additional meloxicam may be used 24 h postoperatively if indicated.

**FIGURE 1:**
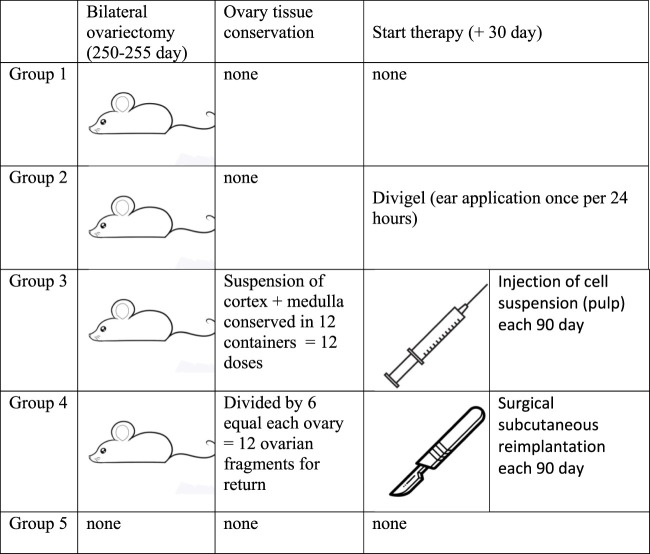
Groups of investigation and research design scheme.

All animals were divided into five groups—five mice in each group—of which four groups were surgically sterilized at the age of 8 months and 2 weeks for our study (Figure 1). In summary, these are the experimental groups:

1. Group of mice that underwent no additional treatment after castration (gonad removal);
2. Group of mice that were given transdermal estrogens (auricular application of Divigel once per 24 h, starting on day 30 after gonad removal);
3. Group of mice that, after castration, were administered subcutaneous injections of defrozen ovarian autologous suspension (cortex + medulla) once every 3 months. The first injection was administered 30 days after gonad removal;
4. Group of mice that, after castration, were subcutaneously transplanted defrozen ovary fragments (25% of ovary volume by one piece) 30 days after gonad removal (cortex + medulla). The first reimplantation was done in a month after castration, followed by reimplantations each 90 days;
5. Group of mice with no manipulations (control of longevity)

The first group did not receive any therapy after sterilization, while the second group received Divigel (transdermal 17-b ethynylestradiol, OrionPharma) once every 24 h (applied on the ears and shaved areas on the back), starting 30 days after gonad removal. The amount of Divigel (transdermal estradiol preparation) we used for hormonal replacement therapy was estimated to be 0.5–0.7 mg, which was equal to 1 g standard recommended dosage for women 65–70 kg weight.

Ovarian tissue was converted into a suspension containing both the cortical and medulla layers and underwent cryopreservation by slow freezing in a xenon medium (purity 99.996%) at a pressure of 1.6 ATM (Shishova and Fesenko, 2015) for subsequent subcutaneous injections in group 3, and the suspension of each individual was divided into 12 portions for further periodical subcutaneous injections. For mice in group 4, the removed ovaries were divided into six parts and also frozen in xenon medium. In group 4, 30 days after castration, a quarter of the thawed ovary was reimplanted subcutaneously in the back region (see the protocol above). Group–3 mice, after castration, were administered subcutaneous injections of the defrozen ovarian autologous suspension (cortex + medulla) once every 3 months. The first injection is done 30 days after gonad removal. Group-4 mice, after castration, were subcutaneously transplanted defrozen ovary fragments (approx. 16% = 1/6 ovary volume by one piece) 30 days after gonad removal (cortex + medulla). The first reimplantation was in a month after castration, followed by reimplantations each 90 days. Group-5 mice with no manipulations (control of longevity), consisting of five mice, served as a control to assess the comparative life expectancy (Figure 1), and hormone levels were measured in the diestrus phase.

In the studied groups, the mice were distributed in separate boxes to exclude the Whitten effect (Gougeon, 1996). The animals were provided with a similar periodic light regime in artificial daylight mode light/dark 16 h/8 h, *ad libitum* nutrition with the same food (protein content 34%, manufactured by Well Plus Trade Vetriebs GmBH, Germany), and were given the same drinking water. The feeding was produced on demand with artificial feed mixtures balanced in terms of proteins, fats, and carbohydrates, identical for each group. Feeding of laboratory animals (mice) was carried out with full-feed granulated feed, made in accordance with the standard “Complete feed for laboratory animals” in accordance with the laws of our country (Mason et al., 2009).

Hormones, including follicle stimulating hormone (FSH), luteinizing hormone (LH), and estradiol, were measured at the Clinical Biochemistry Department at our university hospital as normal routine samples. FSH, LH, and estradiol levels were measured via the Roche Elecsys E2G (FSH) 300, Elecsys Estradiol 3, Elecsys LH (LH) on electrochemiluminescence immunoassay “ECLIA” intended for use on cobas e immunoassay analyzers (United States).

Statistical processing of the results was performed using Stata software (version 3, StataCorp). Data are presented as the M ± SE, Kaplan–Meier, with 99% confidence interval. Significance of differences was evaluated according to U criterion Mann–Whitney *U = nx ∙ ny + (n+1)/2 − T*, and the confidence interval was calculated for the probability of *p* = 0.95 and 0.99.

## Results and discussion

The level of hormones in the blood of mice during the experiment was estimated after castration in group 1, in comparison with the control. A twofold decrease in the serum estradiol level was observed by 15 days after gonad removal. This rate of decrease in estradiol levels corresponds to that in women (1,972 and 342 pg/mL) if castrated during surgeries (e.g., onco). A gradual increase in the FSH level was recorded after 2 weeks, but by 30 days, it was already statistically significant (15 and 0.18 mIU/mL; *p* < 0.001). In the remaining groups, prior to the start of “return” therapy, the same changes were recorded, unless the replacement therapy had started. In any way, castration led to equal changes in all groups, except the control.

However, the dynamics of changes in the hormonal profile with the beginning of therapy had its own characteristics in different groups. A higher rate of normalization of estradiol levels and a decrease in FSH and LH levels were noted with the subcutaneous administration (autotransplantation) of a thawed suspension of the ovarian tissue (Table 1). Then, 2 weeks after the first injection, the level of estradiol increased to 2,000 pg/mL and higher, and after reimplantation of fragmented ovary pieces, only to day 60. This difference is explained by the fact that the speed of revascularization for future functioning of a larger fragment is much lower than that with the introduction of a fragmented suspension. Probably, the tissue suspension has more free estradiol than larger fragments. Nevertheless, in order to compare the effectiveness of one and the second method under identical conditions, we followed the same stages of reimplantation in groups 3 and 4 once every 90 days. In all cases, after the natural death of mice, at the site of reimplantation of an ovarian fragment or injection of an ovarian suspension, we found signs of vascularization in the subcutaneous layer of cortical and medullary fragments. Thus, the injection of a tissue suspension also leads to the long-term functioning of autologous tissues. In both groups 3 and 4, we noted a significant decrease in FSH levels in 1 month, which is the most reliable marker of ovarian deficiency elimination: 0.6 and 1.8 mMU/mL. Even the administration of transdermal estradiol did not cause a rapid effect—the FSH levels decreased by 8 mMU/mL in 30 days after first estradiol application (Table 2).

**TABLE 1:** Hormone levels in mice at different experiment stages.

	Estradiol (pg/mL)	FSH (mMU/mL)	LH (mMU/mL)
Group 1 (castrated) 15 days after gonad removal	1979 ± 320	2, 4 ± 1, 2	2, 8 ± 1, 3
Group 1 (castrated) 30 days after gonad removal	342 ± 15	15 ± 2	17 ± 6
Group 2 (castrated + Divigel) 30 days after first estradiol application	3,576 ± 445	8 ± 1	10 ± 2, 4
Group 3 (castrated + suspension reimplantation) 15 days after first injection of tissue suspension	2,345 ± 210	16 ± 3	14 ± 2
Group 3 (castrated + suspension reimplantation) 30 days after first injection of tissue suspension	3,245 ± 316	0, 6 ± 0, 2	0, 4 ± 0, 2
Group 4 (castrated + 25% ovary volume reimplantation) 15 days after first reimplantation	1,312 ± 450	3, 7 ± 1, 1	3, 4 ± 0, 6
Group 4 (castrated + 25% ovary volume reimplantation) 30 days after first reimplantation	1,643 ± 290	7, 6 ± 3, 4	11, 4 ± 1, 1
Group 4 (castrated + 25% ovary volume reimplantation) 60 days after first reimplantation	2,854 ± 755	1, 8 ± 0, 4	1, 1 ± 0, 3
Group 5, control group of intact mice	3,156 ± 288	0, 18 ± 0, 11	0, 21 ± 0, 08

**TABLE 2:** Mouse longevity at different experimental stages.

	Average longevity, days	Age at gonad removal, days	Age at the beginning of treatment (transdermal hormones, transplantation autologous)	Longevity after beginning therapy, days
Group 1 (castrated)	420 (362–441)	250–255	—	—
Group 2 (castrated + Divigel)	641 (588–645)	250–255	280–285	360
Group 3 (castrated + suspension reimplantation)	930 (796–931)	250–255	282–285	645
Group 4 (castrated + 25% ovary volume reimplantation)	924 (861–952)	250–255	280–285	639
Group 5—control	623 (565–640)	—	—	—

For groups 4 and 5 and groups 3 and 5, *p*-value <0.001.

**FIGURE 2:**
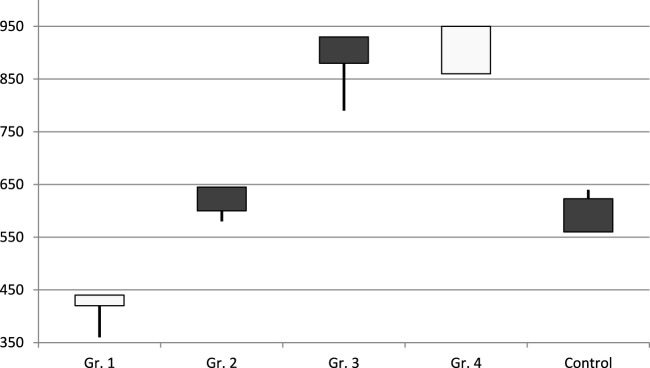
Lifespan of mice in different groups (days) (original by author).

**FIGURE 3:**
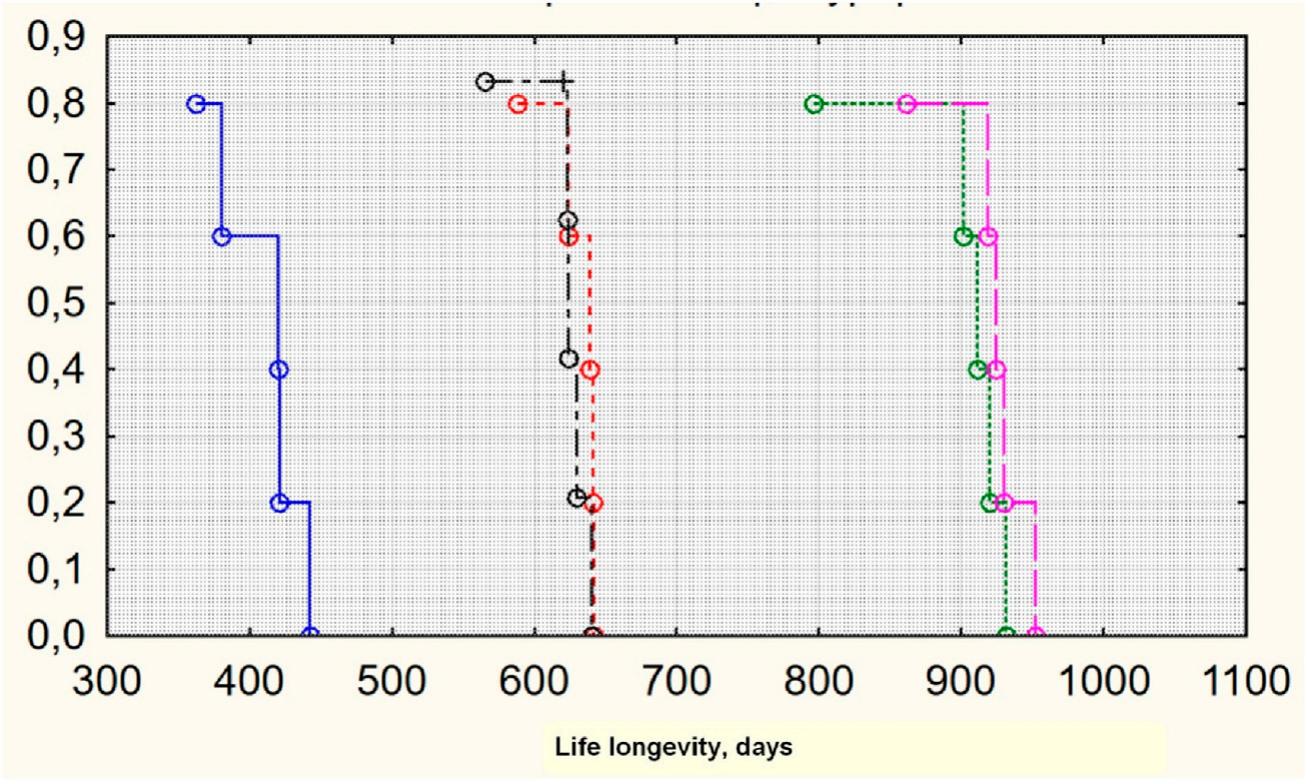
Survival Kaplan–Meier curves for groups of investigation (lifespan). From left to right: groups 1, 5 (control), 2, 4, and 3 (original by author).

The MHT effect of both exogenic estradiol administration and autotransplantation technique led to the best lifespan (Figure 2). The maximum Kaplan–Meier survival age was in groups 3 and 4, where the transplantation was performed (930 and 924 median days, respectively) (Figure 3). Castration alone, with no MHT, resulted in the shortest lifespan—420 median days. Transdermal estradiol administration improved the lifespan to 641 (588–645) median days (*p* < 0.001).

Estradiol levels and decreased FSH and LH levels were not predictors of the lifespan of individuals. Despite the highest speed and effectiveness of replacing estradiol deficiency in the blood by its transdermal use in group 2, we did not note a significant increase in the life expectancy of animals. It really did not differ significantly from the 5th control group (641 and 623 days, respectively; *p* > 0.05), but it was 30%–40% lower than when using periodical autotransplantation of the complex of ovarian tissues (Isachenko et al., 2016; Mason et al., 2009). Consequently, the periodical transfer of the complex of ovarian tissues (cortex + medulla) results in an increase in the life span of individuals since it “protects” the ovarian tissue from physiological exhaustion. The medullary component of the ovary leads to better survival of ovarian tissues during conservation, as confirmed by Isachenko et al. (2016). The key role, according to the authors, is played by phosphatidylserine, a phospholipid, which plays a signaling function in the activation of apoptosis, and its release from the cortex + medulla complex is significantly lower than with the conservation of only the cortical layer (Candy et al., 2000; Soleimani et al., 2011b).

Our study is consistent with the work of Benedusi V, Martini E, Kallikourdis M (2015), who showed a decrease in the life expectancy with a decrease or complete absence of ovarian activity (Benedusi et al., 2015). Estrogens have an anti-inflammatory effect, thereby reducing the progression of chronic inflammation. However, hormone replacement therapy does not have the desired effect compared to full-fledged ovarian tissue, which suggests that biologically active substances with anti-inflammatory effects are produced in the ovaries.

Indeed, *in vitro* estrogens act as antioxidants (Soleimani et al., 2011b). However, with their low plasma concentration in the organism as a whole, they are unlikely to directly have such an effect *in vivo*. Nevertheless, estrogens cause a pronounced antioxidant effect. After an ovariectomy, the production of H_2_O_2_ by mitochondria increases by 50%. This can be prevented by the introduction of estradiol and the transplantation of ovarian tissue (Poirot et al., 2012). Incubation of cells containing the estrogen receptor MCF7 with estradiol significantly reduces the rate of H_2_O_2_ production. The antioxidant effect of estrogen is mediated by its receptor (Shikanov et al., 2011). It turned out that the mitochondria of females produce approximately half the amount of H_2_O_2_ observed in males. Apparently, this is due to the fact that the active substances of the medullary layer have a higher activity of superoxide dismutase and glutathione peroxidase due to the induction of mitosis-activated protein kinases (MAPs) and nuclear transcription factor NF-kB, which trigger the transcription of antioxidant enzymes. The phased replacement of ovarian tissue allows us to activate these processes in which estradiol does not play a major role (Stoop et al., 2014).

## Conclusion

In the current study, we observed the improvement in life longevity in mice after autologous periodical ovarian transplantation after primary castration. The model we suggested could be extended to humans as the cortical transplantation provides the same results for reproduction in mice and humans, but there is still a lack of information about anti-aging factors in the ovarian medullas, which is why we consider the most important factor for anti-aging transplantation technology is to preserve both the medulla and cortex, which leads to a hormonal triggering effect after autologous tissue return.

Our study indicates the important role of medullary ovarian factors in slowing the aging process of the body and increasing the life expectancy in the experiment. As shown, transdermal estrogen supportive therapy in ovarian deficiency improves estrogen levels but causes much slower decreases in FSH and LH levels. Moreover, we attained the best longevity in the experiment with step-by-step periodic ovarian autotransplantation, thus making the “prosthetics” of ovarian function longer than it is preplanned physiologically [direct correlation between the levels of FSH and lifespan (r = 0.98)].

The improvement in lifespan could be reached by means of repeated (once 3–9 months) autologous return of ovarian tissue, preserving low FSH levels as a marker of sufficient ovarian activity. The longevity effect of ovarian transplantation is obvious, while there is lifespan improvement while using traditional menopausal hormonal therapy. Further investigations on the dynamics of direct endocrine and triggering effects of transplanted ovarian fragments should be performed prospectively.

## Summary

Step-by-step autologous ovarian transplantation provides an improvement in life longevity in female mice. There is more efficiency compared to estrogen hormonal replacement therapy in hormone level improvement (FSH and estradiol). While MHT does not improve longevity in mice, step-by-step autologous transplantation of ovarian cryopreserved tissue statistically prolongs the lifespan in mice.

## Data Availability

The raw data supporting the conclusion of this article will be made available by the authors, without undue reservation.
